# Supplementary material for: Contagion dynamics in self-organized systems of self-propelled agents
Source: Sci Rep. 2022 Feb 16;12:2588. doi: 10.1038/s41598-022-06083-0 (PMC8850614; doi:10.1038/s41598-022-06083-0)
Supplement: Supplementary file 1 — Supplementary Information. [file 41598_2022_6083_MOESM1_ESM.pdf]

Supplementary Information:

Contagion dynamics in self-organized systems
of self-propelled agents

Yinong Zhao, Cristián Huepe, and Pawel Romanczuk

1 Mean-field approach for contagion in disordered states

In this section, we use a mean-field approach to carry out a semi-analytical calculation of the contagion in the disordered states described in the main paper, and compare the results to numerical simulations.

The disordered states considered here are similar to those displayed by systems of active Brownian particles. The epidemic spreading in those systems has been previously studied in the literature[1–3], where it was shown that it can be well described by the mean-field approximation. These results cannot be directly applied to our study, however, since they consider different epidemic models and regimes. The analyses in[1,2] examine a Susceptible-Infectious-Susceptible (SIS) contagion process that develops either in low-density systems, where most collisions are binary, or in high-density systems, where agents form an approximately hexagonal lattice. The study in[3] analyzes SIS and SIR infection processes that are instantaneous (in the limit of immediate contagion), have a long infection duration (in the SIR case) and also develop in the low-density regime.

Here, we estimate the eventual recovered fraction following the same type of approaches developed in these references, but applied to the contagion process and regimes considered in our paper. We thus compute the effective rate $\tilde{\beta}$ at which a single infectious agent infects others in a completely susceptible population.

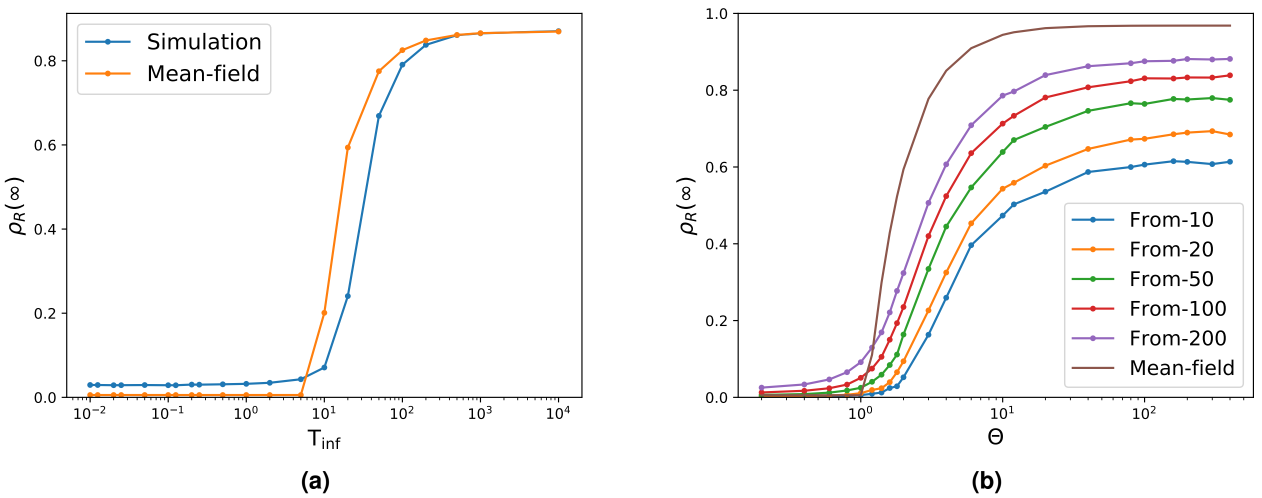

Figure 1. The comparison between numerical simulation and mean-field approach for the representative disordered state. (a) The eventual recovered fraction $\rho_R(\infty)$ as a function of T_{inf}, with the infection-lifetime transmissibility $\Theta = \beta_b/\gamma = 2$ fixed and 100 random agents initially infected, namely $\rho_I(0) = 0.01$. (b) The eventual recovered fraction $\rho_R(\infty)$ as a function of Θ, with infection duration $T_{inf} = 20$ fixed. Results for different initial number of infected agents (from 10 to 200 out of $N = 10000$) is plotted together with mean-field prediction. Other simulation parameters as the representative disordered state in the main text.

$$\tilde{\beta} = \psi(\rho, v_0, \sigma, \beta_b)\nu(\rho, v_0, \sigma) \tag{1}$$

where ν denotes the rate of an agent to contact other agents, which is determined by the parameters of the movement model, including density ρ, speed v_0, angular noise σ (τ is neglected because alignment strength is very weak); ψ denotes the probability of an infected agent infecting a susceptible agent during the contact with it, which is determined by β_b together with movement parameters. We have

$$\psi(\rho, v_0, \sigma, \beta_b) = \int_0^\infty \mathrm{d}\omega p(\omega)(1 - e^{-\omega\beta_b}) \tag{2}$$

as in[2], where ω denotes the duration of two particles stay in contact, $p(\omega)$ denotes the probability distribution of ω, which is determined by v_0, σ and ρ. Using numerical simulations, we can empirically calculate $\nu(\rho, v_0, \sigma)$ and $\psi(\rho, v_0, \sigma, \beta_b)$ with β_b given, and hence the basic reproduction number

$$\mathrm{R}_0 = \frac{\tilde{\beta}}{\gamma} , \tag{3}$$

which determines the final recovered fraction in the conventional SIR model. In particular, for the case $\beta_b \to 0$ (or $\mathrm{T_{inf}} \to \infty$ while Θ fixed), $\psi = \int_0^\infty \mathrm{d}\omega p(\omega)\omega\beta_b = \langle\omega\rangle\beta_b$, $\mathrm{R}_0 = \nu\langle\omega\rangle\Theta$, where ν is the average contact rate and $\langle\omega\rangle$ is the average contact duration, namely, the basic reproduction number in high $\mathrm{T_{inf}}$ case is proportional to the infection-lifetime transmissibility Θ. For the representative disordered state ((Pe,g)=(32,1)), we compare the mean-field predictions and average numerical results in Fig. 1.

As shown in the Fig. 1a, our simulation result matches asymptotically the mean-field prediction at the high $\mathrm{T_{inf}}$ limit. With $\mathrm{T_{inf}} = 20$ fixed, our simulation and mean-field prediction do not match (as shown in Fig. 1b with red curve for numerical simulation with 100 initial infected agents and the brown curve for mean-field approach). Mean-field approach assumes that agents in different contagion states are well mixed. However, this is not the case for small $\mathrm{T_{inf}}$, where infection dies out before sufficient mixing, which leads to the deviation of simulation result from mean-field predictions (as $10 \leq \mathrm{T_{inf}} \leq 40$ in Fig. 1a and the whole Fig. 1b). As mixing is effectively enhanced by increasing the initial number of infected agents, the simulation results progressively match the mean-field prediction (see Fig. 1b), but the increasing initial infection also increasingly leads to deviations for subcritical parameter values, e.g. small Θ (c.f. small $\mathrm{T_{inf}}$ or Θ in Fig. 1a and Fig. 1b). To accurately predict the eventual recovery, active diffusion and other properties of the system need to be taken into account, which is beyond the scope of this study.

2 Representative time series of constitutions

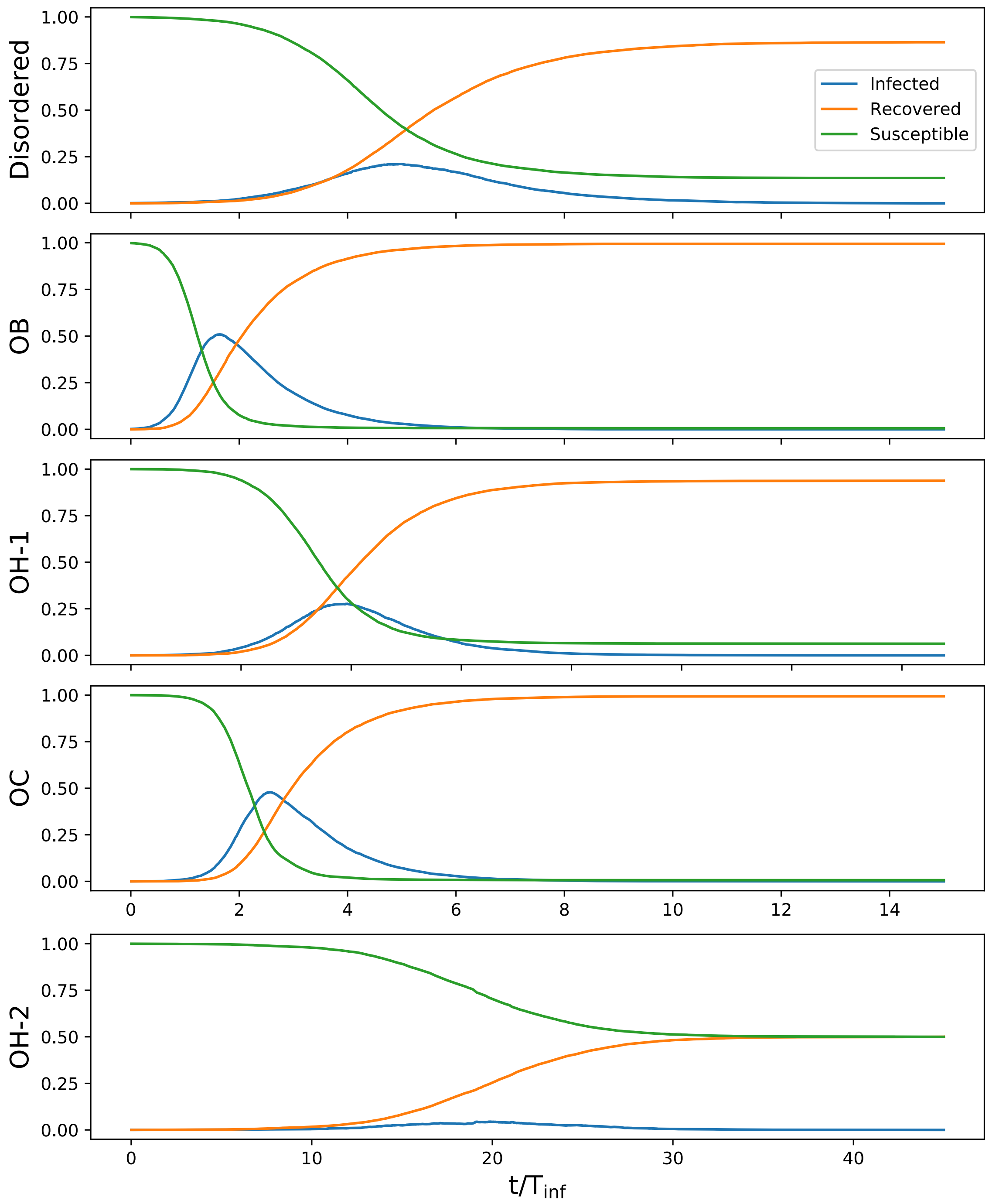

Figure 2. Typical evolution of the fractions of infected agents, recovered agents, and susceptible agents as a function of time in the five different states considered in the main text using the same simulation parameters (OH-1: (Pe,g)=(512,16); OH-2: (Pe,g)=(32,1024); OB: (Pe,g)=(32,2); OC: (Pe,g)=(32,128); Disordered states: (Pe,g)=(32,1)) for $T_{Inf} = 10^4$ and $\Theta = 2$ (cf. Fig 3b in the main text). Except for state OH-2, all time series share a same time axis, which is indicated in the x-axis of OC state; for the state OH-2, the time axis is indicated separately. As described in the main text, OB and OC states display the fastest contagion dynamics while state OH-2 exhibits the slowest.

3 Simulation videos

To better illustrate the contagion processes in the different states described in the main paper, we provide representative videos of the dynamics of each case.

The Simulation videos include the OH-1 , OH-2, OB, OC, and disordered states, simulated with the same movement parameters as in the main text (see also previous section). The contagion parameters are set to $T_{inf} = 10^3$ and $\Theta = 2$. For all movies, we initially infect a small group of agents in the center of the arena.

The filenames and descriptions of all simulation videos are the following:

- **Supplementary Video 1 - Disordered state:** Example contagion process in the disordered state in the absence of orientational order. We only include the period from the initial infection to a significant outbreak.
- **Supplementary Video 2 - Ordered homogeneous state 1:** Example of infection spreading in the form of a band in the ordered homogeneous state OH-1, as described in the main text. We start the clip when an apparent infection band is formed.
- **Supplementary Video 3 - Ordered homogeneous state 2:** Example of the contagion dynamics in the ordered homogeneous state OH-2. In the displayed regime, the initial infection rarely leads to an outbreak, but we selected a specific case where a small outbreak develops to illustrate how the contagion process can happen in the OH-2 state. The clip starts from a period after the initialization, when the contagion appears clearly in a cluster. We can observe how the cluster collapses and spreads the infection, forming an infected front that is elongated perpendicular to the heading direction.
- **Supplementary Video 4 - Ordered band state:** Example of contagion dynamics in the ordered state with moving band.
- **Supplementary Video 5 - Ordered cluster state 1:** Example of the initial infection spreading within a cluster in the ordered clustered state OC.
- **Supplementary Video 6 - Ordered cluster state 2:** Example of a later stage of contagion in the ordered clustered state, where the contagion between clusters is driven by fission-fusion dynamics.

References

1. Peruani, F. & Sibona, G. J. Dynamics and steady states in excitable mobile agent systems. *Phys. review letters* **100**, 168103 (2008).

2. Peruani, F. & Sibona, G. J. Reaction processes among self-propelled particles. *Soft matter* **15**, 497–503 (2019).

3. Norambuena, A., Valencia, F. J. & Guzmán-Lastra, F. Understanding contagion dynamics through microscopic processes in active brownian particles. *Sci. Reports* **10**, 1–7 (2020).
